# Mechanisms of Autoantibody Production in Systemic Lupus Erythematosus

**DOI:** 10.3389/fimmu.2015.00228

**Published:** 2015-05-13

**Authors:** Shuhong Han, Haoyang Zhuang, Stepan Shumyak, Lijun Yang, Westley H. Reeves

**Affiliations:** ^1^Division of Rheumatology and Clinical Immunology, University of Florida, Gainesville, FL, USA; ^2^Department of Pathology, Immunology, and Laboratory Medicine, University of Florida, Gainesville, FL, USA

**Keywords:** autoantibodies, lupus erythematosus, systemic, innate immunity, TLR7, B cells, immune tolerance

## Abstract

Autoantibodies against a panoply of self-antigens are seen in systemic lupus erythematosus, but only a few (anti-Sm/RNP, anti-Ro/La, anti-dsDNA) are common. The common lupus autoantigens are nucleic acid complexes and levels of autoantibodies can be extraordinarily high. We explore why that is the case. Lupus is associated with impaired central or peripheral B-cell tolerance and increased circulating autoreactive B cells. However, terminal differentiation is necessary for autoantibody production. Nucleic acid components of the major lupus autoantigens are immunostimulatory ligands for toll-like receptor (TLR)7 or TLR9 that promote plasma cell differentiation. We show that the levels of autoantibodies against the U1A protein (part of a ribonucleoprotein) are markedly higher than autoantibodies against other antigens, including dsDNA and the non-nucleic acid-associated autoantigens insulin and thyroglobulin. In addition to driving autoantibody production, TLR7 engagement is likely to contribute to the pathogenesis of inflammatory disease in lupus.

The production of autoantibodies, in particular antinuclear antibodies (ANA), is nearly universal among patients with systemic lupus erythematosus (SLE). Autoantibodies against a wide diversity of cellular antigens (180 by one count) have been reported (1). However, the common autoantibody “phenotypes” in this disease are not nearly as diverse as suggested by the list of known autoantibodies. We argue that abnormal B-cell censoring accounts for the diversity of autoantibodies that can be detected in SLE patients’ sera by sensitive techniques such as ELISA, whereas the highly restricted list of commonly produced, high-level autoantibodies is a consequence of innate immune recognition of autologous (immunostimulatory), nucleic acids associated with these antigens.

## Multiple Mechanisms Limit Autoantibody Production

Positive and negative signals derived from the B-cell receptor (BCR) and co-receptors along with competition for survival factors such as B-cell activating factor (BAFF, also known as BLyS), determine whether a B-cell is activated, deleted, or rendered anergic (2, 3). Regulatory T cells play an additional role in determining the outcome of positive and negative signals. Genetic abnormalities affecting BCR signaling can lead to increased numbers of circulating autoreactive B cells.

The early B-cell repertoire in the bone marrow (BM) consists of many BCRs displaying affinity for self-antigens, such as DNA. B cells expressing receptors with high affinity for self-antigens expressed in the BM typically are deleted before they can exit the BM or else undergo receptor editing to generate a receptor without self-reactivity. However, B cells that bind self-antigens weakly or recognize self-antigens not found in the BM can exit to the periphery, where they are censored by deletion or anergy-induction. A second wave of autoreactive B cells is generated peripherally in germinal centers (GC) as a consequence of somatic hypermutation of the immunoglobulin heavy and light chain variable regions. A single mutation in one of these regions can convert a BCR with specificity for a bacterial antigen (phosphorylcholine) into a receptor with specificity for DNA (4). About 40% of immature, newly emigrated peripheral blood B cells from healthy individuals are autoreactive as determined by HEp-2 cell lysate ELISA (5). However, these cells are subject to censoring between the immature and naïve B-cell stages of development and do not secrete immunoglobulin unless they undergo further maturation and terminal differentiation into plasma cells. Thus, in addition to the censoring (central and peripheral) of autoreactive B cells, regulation of terminal B-cell differentiation may limit production of serum autoantibodies.

## Abnormal Censoring of Autoreactive B Cells in SLE

B cells from patients with SLE and mice with lupus-like disease exhibit abnormalities that alter the strength of BCR signaling and increase the generation of autoreactive B cells due to impaired central and/or peripheral censoring. Examples include abnormalities affecting *PTPN22*, Bruton’s tyrosine kinase (*Btk*), and *Lyn*, and BAFF (*Tnfsf13b*).

### PTPN22

Polymorphism of the protein tyrosine phosphatase *PTPN22* is associated with several autoimmune disorders characterized by autoantibody production, including SLE, Type 1 diabetes (T1D), rheumatoid arthritis, Grave’s disease, and myasthenia gravis (6). The C1858T polymorphism decreases B-cell responsiveness, impairing central deletion and editing of autoreactive B cells, leading to autoantibody production (6, 7).

### Bruton’s tyrosine kinase

Transgenic expression of *Btk* in mice impairs both central and peripheral B-cell tolerance by altering the threshold for B-cell activation and reducing negative selection of autoreactive B cells (8). BCR hyper-responsiveness in these mice promotes spontaneous GC formation, a common finding in lupus-like autoimmune syndromes, as well as the production of ANAs and development of renal, lung, and salivary gland pathology.

### Lyn

Mice with B-cell-specific deficiency of the tyrosine kinase *Lyn* develop anti-dsDNA and anti-Sm autoantibodies and nephritis. SLE patients with low LYN expression also have been reported (9). Due to enhanced signaling in transitional and follicular B cells and increased levels of BAFF, *Lyn*-deficient mice have abnormal peripheral censoring and develop increased numbers of B1a cells with abnormal GC formation (10). Autoimmunity is abolished by deficiency of the adapter protein MyD88, suggesting that toll-like receptors (TLRs) are involved.

### B-Cell activating factor

Expression of transgenic *Tnfsf13b* (encoding BAFF) in mice alters peripheral B-cell tolerance. Follicular and marginal zone B cells compete for a limiting amount of this B-cell survival factor, and increased BAFF levels allow autoreactive B cells to escape censoring, resulting in increased GC formation, autoantibody production, and the development of glomerulonephritis (11, 12). Increased BAFF levels also are seen in SLE patients (13). Autoantibody production in BAFF-transgenic mice is T-cell-independent and MyD88/TLR-dependent (14).

## Regulation of Terminal B-Cell Differentiation

B-cell signaling defects affecting central or peripheral tolerance increase the number of circulating autoreactive B cells, but not necessarily their maturation into autoantibody-secreting plasma cells. The restriction of terminal B-cell differentiation may limit autoantibody production once autoreactive B cells have escaped censoring. Plasma cell differentiation is controlled by two sets of mutually antagonistic transcription factors. The B-cell gene expression program is maintained by PAX5 and BCL-6, whereas BLIMP-1 (*PRDM1* gene) and XBP1 promote the generation and survival of plasma cells (15). PAX5 and BCL-6 repress *PRDM1* and PAX5 also represses *XBP1*. Conversely, BLIMP-1 represses *BCL-6* and *PAX5*. In GCs, B-cell activation via the BCR, T cells (CD40–CD40L interactions), and/or TLR signaling causes NF-κB-mediated up-regulation of IRF4, which induces *PRDM1* while repressing *BCL-6*. IRF4 also is up-regulated by BAFF-stimulated NF-κB activation, a potentially important pathway in BAFF-transgenic mice (14).

The transcription factor STAT3 also up-regulates *PRDM1* expression and is activated by the cytokines IL-10, IL-21, IL-6, and IFNα (15). T-cell-dependent terminal differentiation of IgG B cells is abolished in STAT3-deficient mice (16). BAFF acts synergistically with MyD88 (TLR) (14) and with IL-21 (17), and TLR7 acts synergistically with T-cell-derived signals (CD40–CD40L and SAP) to drive plasma cell differentiation (18).

### Long- vs. short-lived plasma cells

Regulation of autoantibody levels also depends on the lifespan of autoantibody-producing plasma cells. Plasma cells can be generated either as a product of GC reactions or extrafollicularly. Both pathways regulate human autoantibody production. GC-derived plasma cells have a propensity to home to the BM where they survive and can secrete immunoglobulin for many years as long-lived plasma cells, whereas plasma cells generated outside of GCs (extrafollicular) usually are short-lived, secreting immunoglobulin for a week or two before undergoing apoptosis. The persistence of high levels of anti-Sm/RNP and Ro/La autoantibodies in SLE is most consistent with a GC-derived long-lived plasma cells and memory B cells. In contrast, the transitory presence of anti-dsDNA autoantibodies during disease flares is most consistent with production of these autoantibodies by short-lived plasma cells. Further, the inconsistent response of anti-dsDNA autoantibody levels to rituximab (anti-CD20 monoclonal antibody) therapy supports the existence of autoantibody-producing short- and long-lived plasma cells (19, 20). In our experience, levels of autoantibodies against RNA-associated autoantigens such as anti-Ro (SS-A) generally have been less responsive to rituximab than anti-dsDNA autoantibodies. Since plasma cells are CD20-negative, the degree to which autoantibody levels decline following rituximab infusion may be an indicator of the relative contribution of short-lived plasma cells to maintaining autoantibody levels.

### Follicular vs. extrafollicular pathways

Germinal center responses are characterized by a requirement for T follicular helper (T_FH_) cells, production of class-switched and somatically mutated antibodies, and generation of long-lived plasma cells and memory B cells. T-cell expression of BCL-6 is critical for both GC and extrafollicular B-cell responses (21). T_FH_ cells (GC pathway) generally are Bcl-6^+^, PD-1^hi^, whereas T cells involved in extrafollicular B-cell responses are Bcl-6^+^, PD-1^lo/int^. An expanded population of circulating PD-1^hi^ T_FH_-like cells is seen in patients with active SLE and is associated with the number of circulating plasmablasts and anti-dsDNA autoantibody positivity; these cells may be a surrogate for aberrant GC activity in SLE (22).

Mice homozygous for a mutation of the ubiquitin ligase *Roquin* (sanroque mice) also accumulate T_FH_ cells (23). IL-21 production by T_FH_ enhances class switching and plasma cell differentiation by increasing the expression of activation-induced cytidine deaminase and BLIMP-1, promoting autoantibody production (24). As GC B cells compete for limited numbers of T_FH_ cells, the increased CD40–CD40L interactions and IL-21 in sanroque mice promote survival of autoreactive B cells and their development into plasma cells.

In contrast to follicular B cells, B-1 and marginal zone B cells preferentially generate short-lived (extrafollicular) plasma cells. Although GC responses generally require CD40–CD40L interactions to initiate plasma cell differentiation, differentiation of human marginal zone-derived memory B cells is CD40-independent, but IL-21 and BAFF-dependent (17).

### Role of TLR in plasma cell differentiation

In mice, endosomal TLRs are critical for the production of lupus autoantibodies. In MRL/*lpr* mice, anti-Sm autoantibodies are abolished in TLR7-deficient mice, whereas anti-dsDNA autoantibodies are absent in TLR9-deficient mice (25). Nevertheless, disease progression is ameliorated by TLR9 in MRL/*lpr* mice. In contrast, both anti-Sm/RNP and anti-dsDNA autoantibodies are TLR7-dependent in pristane-induced lupus (26). Production of anti-Sm/RNP autoantibodies is driven by dual engagement of the BCR (by the protein components of U1 snRNPs) and TLR7 (by U1 RNA) (27). TLR signaling in memory B cells may play a role in maintaining serological memory by driving their terminal differentiation (28), though the role of B-cell-intrinsic, polyclonal TLR signaling in maintaining antigen-specific antibody levels is controversial (29), possibly because the requirement for TLR signaling may differ depending on the nature of the antigen. This is illustrated by antibody responses to virus-like particles (VLPs). Using a soluble TLR9 ligand as adjuvant, dendritic cell but not B-cell MyD88 expression is needed to enhance antibody production. In contrast, when the TLR9 ligand is incorporated into a VLP, B-cell-intrinsic expression of TLR9/MyD88 is required (30, 31). This may reflect enhanced delivery of nucleic acid to endosomes containing TLR9. Since their nucleic acid constituents are less susceptible to endosomal degradation when associated with proteins, DNA-protein (e.g., chromatin) and RNA-protein (e.g., Sm/RNP, U1 snRNPs) autoantigens may resemble VLPs.

Ligands for the endosomal TLRs (TLR3, TLR7, TLR8, TLR9) are potent adjuvants (32). The pro-inflammatory effects of TLR signaling play a role in their “adjuvanticity.” TLR7 is encoded on the X-chromosome, one copy of which is inactivated in female cells. Male BXSB (*Yaa*) mice, which have a second active copy of the *Tlr7* gene on the Y chromosome, develop a spontaneous lupus syndrome with increased IL-21 and anti-dsDNA autoantibody production, and glomerulonephritis (33). In B6 mice, levels of anti-RNA autoantibodies are correlated with the *Tlr7* copy number (34).

In addition to the level of TLR expression, subcellular distribution is critical for promoting inflammatory responses to foreign nucleic acids and preventing inappropriate responses to autologous nucleic acids. TLR7 and TLR9 remain inactive until they are proteolytically cleaved inside acidic endosomes. The endoplasmic reticulum-resident protein UNC93B1 controls intracellular trafficking of endosomal TLRs, regulating their activity and helping to prevent recognition of endogenous nucleic acids. By competing with TLR7 for UNC93B1, TLR9 plays an anti-inflammatory role, as illustrated by mutations that abolish the preference of UNC93B1 for TLR9, resulting in lethal TLR7-mediated inflammatory disease (35, 36).

## Levels of Autoantibodies Against Diverse Self-Antigens in SLE Patients

Titers of IgG autoantibodies against RNA-protein autoantigens, such as U1 snRNPs can be as high as 1:10^6^ and these levels typically are maintained for many years (37). This is much higher than reported in other autoimmune diseases or following immunization with exogenous foreign antigens. An important difference between autoantibody responses in SLE vs. those in other disorders, such as T1D or autoimmune thyroid disease, is the physical nature of the autoantigens. Autoantibodies in T1D recognize pancreatic antigens, such as insulin, glutamic acid dehydrogenase, and Pdx-1, which are not associated with nucleic acids. These autoantibodies are specific for T1D, just as anti-Sm and anti-dsDNA autoantibodies are specific for lupus. But diabetes-specific autoantibodies typically are reported at much lower levels than anti-Sm/RNP (38). We hypothesize that the explanation for extremely high titers of “classic” lupus autoantibodies such as anti-Sm, RNP, Ro (SS-A), and La (SS-B) is their association with endogenous adjuvant, in the form of nucleic acid. Other autoantibodies reported in SLE (1) do not recognize nucleic acid-protein antigens. We hypothesized that the latter may be produced at lower levels because they are not associated with endogenous adjuvant.

**Figure 1:**
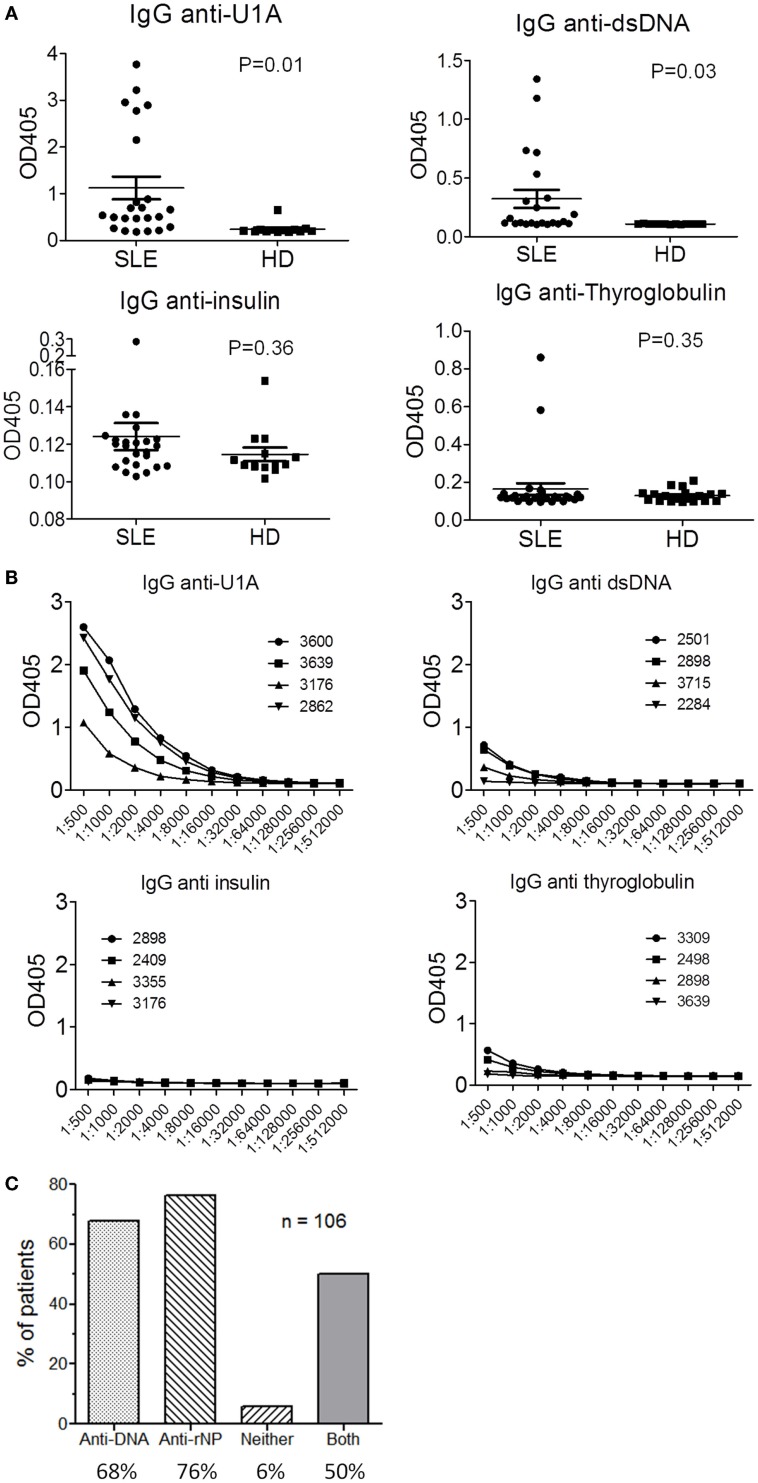
**Serum autoantibodies in SLE**. Levels of serum IgG anti-U1A, dsDNA, insulin, and thyroglobulin autoantibodies were tested by ELISA in 23 consecutive SLE patients and 13 controls. SLE was classified using the ACR criteria. Healthy controls age and gender-matched had no history of systemic autoimmune disease. These studies were reviewed and approved by the University of Florida Institutional Review Board. **(A)** Antigens were coated on plastic wells at 0.8 μg/ml and sera were diluted 1:500 for screening. **(B)** ELISA titration curves (serial twofold dilutions) for the four sera most strongly reactive with each antigen. Patient ID number is shown in the key. **(C)** Frequencies of anti-dsDNA, anti-RNA-protein (rNP, anti-Sm, RNP, Ro/SS-A, and La/SS-B) autoantibodies detected by commercial ELISAs in a cohort of 106 SLE patients. Only 6% of the sera were unreactive with both dsDNA and the ribonucleoprotein autoantigens.

To test this hypothesis, we examined autoantibody levels in sera from 23 consecutive SLE patients seen in our Autoimmune Disease Clinic and 13 healthy controls (Figure 1). ELISA wells were coated with four test antigens at 0.8 μg/ml: recombinant human U1A (a component of U1 snRNPs), double-stranded (ds) DNA, human insulin, and human thyroglobulin. A positive reaction was defined as ≥2 standard deviations above the mean of controls. In this cohort, 18/23 SLE patients had anti-U1A autoantibodies vs. 1/13 controls (at a low level) (*P* = 0.01) (Figure 1A). For anti-dsDNA antibodies, 7/23 SLE patients vs. 0/13 controls were positive (*P* = 0.03). In the case of anti-insulin autoantibodies, 1/23 SLE patients and 0/13 controls were positive (*P* = 0.36, NS), and the levels of autoantibodies were considerably lower than anti-U1A or anti-dsDNA. Similarly, 2/23 SLE patients vs. 0/13 controls were positive for anti-thyroglobulin autoantibodies (*P* = 0.35, NS). Consistent with the wide diversity of autoantibodies in SLE (1), we identified patients whose sera were positive for anti-insulin autoantibodies (associated with T1D) and anti-thyroglobulin autoantibodies (associated with autoimmune “Hashimoto” thyroiditis). However, these patients did not have either T1D or hypothyroidism. Serum from one patient (#2898) gave a positive reaction with all four self-antigens, suggesting that it may contain “polyreactive” IgG autoantibodies.

We next examined the titers of autoantibodies against each test antigen in the four most strongly positive SLE sera. As shown in Figure 1B, titers of autoantibodies against the RNA-protein autoantigen U1A were markedly higher than those against dsDNA and the non-nucleic acid associated autoantigens insulin and thyroglobulin. Additionally, anti-dsDNA titers were higher than anti-insulin and anti-thyroglobulin.

Finally, we examined the frequency of anti-dsDNA and anti-ribonucleoprotein (Sm, RNP, Ro/SS-A, or La/SS-B) autoantibodies by commercial ELISA in a cohort of 106 SLE patients meeting the Revised ACR Criteria for Classification of Lupus. In these patients, 68% were positive for anti-dsDNA at some time during their course, 76% for anti-ribonucleoprotein, and 50% for both anti-dsDNA and anti-ribonucleoprotein autoantibodies (Figure 1C). Only 6% of the patients did not have anti-DNA-protein or RNA-protein autoantibodies, a figure that may over-estimate the true number, as we did not include autoantibodies against RNA-protein autoantigens other than anti-Sm/RNP/Ro/La.

Taken together, these data suggest that (1) SLE is characterized by a high frequency of autoantibodies against nucleic acid-protein autoantigens, whereas frequencies of autoantibodies against the non-nucleic acid-associated autoantigens are considerably lower and (2) in SLE patients, the levels of autoantibodies against nucleic acid autoantigens (particularly RNA-proteins) is markedly higher than autoantibodies against non-nucleic acid associated autoantigens. Finally, although more speculative, the subset of non-nucleic acid associated autoantibodies (e.g., anti-insulin, anti-thyroglobulin) may be of limited clinical significance, as none of the patients had either T1D or autoimmune thyroiditis. However, we cannot exclude the possibility that they will develop T1D or thyroid disease in the future. In contrast, anti-RNP/Sm autoantibodies (such as anti-U1A) are implicated in the production of pro-inflammatory cytokines, such as IFNα, by plasmacytoid dendritic cells (39) and anti-dsDNA autoantibodies can cause lupus nephritis (40).

## Conclusion

**Figure 2:**
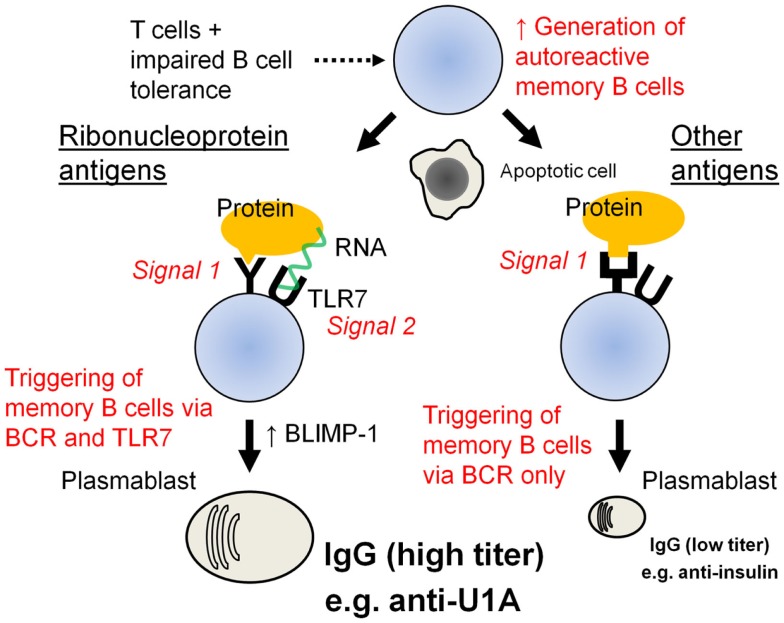
**Proposed model of autoantibody generation in SLE**. SLE is associated with genetic abnormalities that impair central or peripheral B-cell tolerance. In the presence of T-cell help, autoreactive B cells that escape censoring may develop into IgG-producing memory B cells. However, these cells can circulate without producing autoantibodies. Following subsequent antigen exposure, the memory cells enhance their expression of BLIMP-1 and develop into autoantibody-secreting plasma cells. In the case standard autoantigens, such as insulin, which engage only the B-cell receptor, the stimulus to undergo terminal (plasma cell) differentiation is weaker than in the case of RNA-associated autoantigens, such as U1A, which can activate terminal B-cell differentiation via both the B-cell receptor and TLR7. This may explain why levels of anti-U1A (RNP) autoantibodies are markedly higher than anti-insulin autoantibody levels.

Although 180 or more self-antigens have been reported as targets in SLE (1), only a few are common. This subset, which includes anti-Sm/RNP, anti-Ro/SS-A, anti-La/SS-B, anti-dsDNA, and several others, consists primarily of nucleic acid (DNA or RNA) associated proteins. Although lupus-like phenomena are associated with genetic abnormalities that impair central or peripheral B-cell tolerance (e.g., *PTPN22*, *Btk*, *Lyn*, *Tnfsf13b*), large numbers of autoreactive B cells can circulate without producing autoantibodies (5). Thus, regulation of the balance between *BCL-6* and *PRDM1* (BLIMP-1) expression may play a role in determining whether or not clinically significant levels of autoantibodies are produced. T-cell-derived signals (CD40L, SAP), cytokines (BAFF, IL-21), and TLRs (especially TLR7), all promote terminal B-cell differentiation by up-regulating the expression of *PRDM1*. We hypothesize that the enhanced survival of autoreactive B cells in SLE in the setting of BCR engagement and sub-optimal levels of T-cell-derived maturation signals or cytokines may explain the wide diversity of autoantibodies detected in SLE (Figure 2). The production of relatively low levels of anti-insulin, anti-thyroglobulin, and anti-dsDNA autoantibodies (Figure 1B) is consistent with that possibility. The repetitive nature of DNA antigen may further drive plasma cell differentiation by enhancing the strength of BCR signaling. In contrast, dual engagement of the BCR and TLR7 by ribonucleoprotein autoantigens may deliver a more potent terminal differentiation signal, explaining the high levels of autoantibodies against ribonucleoprotein antigens (Figure 2). TLR7 engagement also may play a key role in the inflammatory manifestations of SLE by driving the production of IFNα, TNFα, IL-12 (IFNγ), and other pro-inflammatory cytokines. Thus, while SLE is characterized by a panoply of autoantibodies, the clinically significant autoantibodies are probably more restricted.

## Conflict of Interest Statement

The authors declare that the research was conducted in the absence of any commercial or financial relationships that could be construed as a potential conflict of interest.
